# The continuing evolution of health promotion

**DOI:** 10.1093/heapro/daab150

**Published:** 2021-12-12

**Authors:** Don Nutbeam, J Hope Corbin, Vivian Lin

**Affiliations:** 1 Professor of Public Health in the School of Public Health, University of Sydney, Sydney, NSW 2006, Australia; 2 Department of Health and Community Studies, Woodring College of Education, Western Washington University, Bellingham, WA 98225, USA; 3 Professor of Practice (Public Health), Faculty of Medicine, University of Hong Kong, Hong Kong SAR, China

The *Ottawa Charter for Health Promotion* provided a template that has fundamentally re-shaped public health practice in the past 35 years (WHO, 1986). The compelling logic of its key strategies—build healthy public policy, create supportive environments for health, strengthen community actions, develop personal skills and reorient health services—now routinely provide a comprehensive and inclusive framework for addressing any major public health challenge.

Profound social and economic changes have occurred and continue to occur since the Charter was written. The globalization of trade has had a major impact on economies and systems of governance that have an effect on the lives of everyone. The internet, mobile communications and digital technologies have opened access to information and services that were unimaginable at that time. Our understanding of the interconnections between environmental change, human health and the health of our planet has undergone a fundamental transformation. More than 50% of the world’s population now live in urban settings. New threats to health have emerged. These include previously unknown infectious diseases—from HIV to the current COVID-19 pandemic—as well as more insidious, progressive changes associated with the emergence of non-communicable diseases in all countries.

Such profound changes have required continuous adaptations to the health promotion strategies established through the *Ottawa Charter*. Since 1986 WHO has organized a series of global conferences that have helped to refine and develop the concept and strategies of health promotion. These conferences have addressed the fundamental social and economic changes indicated above and have advocated practical public health responses that have their roots in the strategies of the *Ottawa Charter*. The most recent of these conferences was in 2016 and produced the *Shanghai Declaration on promoting health in the 2030 Agenda for Sustainable Development* (WHO, 2016). The conference positioned health promotion methods and strategies at the heart of actions required to achieve the UN Sustainable Development Goals (SDGs) (United Nations, 2015). At the core of the *Shanghai Declaration* are key strategies advocating improved governance for health, the development of healthy cities and improvements in health literacy in populations around the world.

A commentary on the *Shanghai Declaration* reports that health promotion offers ‘practical solutions to the complex and interconnected challenges represented by the SDGs’, noting that promoting health should become ‘a cornerstone of all national or local SDG strategies and implementation plans’ (Kickbusch and Nutbeam, 2017). The authors note that these challenges can take us to uncomfortable and messy places where health is a politicized concept and not always given the priority that we may wish it to have; and where health promotion is being interpreted in different ways for different purposes. The authors remind us that our role is to demonstrate how health promotion can be delivered most effectively in variable circumstances, and how health promotion ethics can be upheld.

This special supplement to *Health Promotion International* has been supported by the World Health Organization to build on the progress and challenges emerging from the Shanghai Conference and provide a platform for discussion at the 10th Global Conference on Health Promotion. The conference reflects WHO’s triple billion agenda, specifically highlighting the role of health promotion in the pursuit of health and wellbeing for all through WHO’s Healthier Populations framework and measurable impacts (WHO, 2019, 2020).

The purpose of the supplement is to provide a collection of papers summarizing the current state of knowledge and to advance thinking on the future of health promotion. The papers were commissioned in the context of WHO’s current priorities, and its contributions to the achievement of the Sustainable Development Goals (SDGs). WHO’s priorities include achieving Universal Health Coverage (UHC): responding successfully to global health emergencies (including COVID-19); and creating healthier populations (WHO, 2020).

The papers cover a wide terrain, reflecting the core elements of the *Shanghai Declaration* by exploring law and urban governance through the paper by Burris and Lin; and the future evolution of health literacy by Sørensen and colleagues (Burris and Lin, 2021; Sørensen, 2021). The paper on governance suggests that regardless of their powers, organizational structures and boundaries, cities have legal levers they can manipulate for health promotion. They can use their legal powers to address the effects of policies that they themselves can control, and they may also have the authority to use the law to address deeper determinants of health to directly and indirectly reduce economic and social inequality. The paper on health literacy builds on the challenge set out in the *Shanghai Declaration* of delivering large-scale societal improvement in health literacy through a systematic approach to capacity development. The paper presents an overarching framework for sustainable change with a range of action areas to stimulate the structural transformation needed at different levels. These include training a health literate workforce and supporting health literate organizations, as well as enabling health literacy-informed technology and innovation.

Other papers examine the application of health promotion strategies in response to public health emergencies through the paper by Corbin and colleagues; and in addressing the commercial determinants of health through a paper by Bettcher and colleagues (Corbin *et al.*, 2021a; McHardy 2021). The paper on public health emergencies, presents five cases studies from the USA, Singapore, Sierra Leone, Kenya and South Africa and highlights several critical success factors. These include co-development of relevant education and communication strategies, amplification of community leadership and mobilization of community members. Drawing on the experience from the case studies, these strategies enable communities to achieve shared goals, assess and adapt to changing context and prepare for future emergencies. The paper on the commercial determinants showcases the *WHO Framework Convention for Tobacco Control* (FCTC) (WHO, 2003) as a working model for preventing harmful industries ‘from wielding influence over the institutions and actors of global and national governance’. The paper examines different strategies for addressing the commercial determinants of health. It recognizes that formal conventions such as the FCTC are one path for impacting commercial determinants, but that we need to deploy a wider range of strategies to respond and adapt to rapidly changing circumstances.

The paper by Hancock and colleagues examines a further key challenge emerging from the task of positioning health promotion methods and strategies at the heart of actions required to achieve the UN SDGs by advancing planetary health promotion through local-level actions (Hancock *et al.*, 2021). The paper provides strong advocacy for health promotion actors to become part of the emerging network of community organizations and individuals working to create sustainable, just and healthy communities.

Finally, we have two papers that explore emerging issues in health promotion examining evolving concepts in health and wellbeing by Corbin and colleagues; and Digital Health Promotion by Lim and colleagues (Corbin *et al.*, 2021b; Koh *et al.*, 2021). The paper on wellbeing situates the concept within the founding documents of WHO and health promotion, explores alternative measurement strategies, briefly presents promising cases of wellbeing policy initiatives and explores points of controversy and tension. The paper concludes by describing how health promotion might contribute to wellbeing policy frameworks that promote the sources of human and planetary thriving through sustainable development.

The paper by Lim *et al.* examines the potential benefits and challenges of digital technology for current and future health promotion strategies. The benefits include expanding access to health information and health-promoting services, as well as lowering the cost of scaling up activities to reach larger populations. It also highlights key challenges including the growth of misinformation and disinformation through digital media; and ensuring widespread accessibility and affordability to digital technology in ways that minimize social inequities.

Taken together this set of papers provides an overview of several key transformations facing the global community and suggests pathways for the application of health promotion methods and strategies to meet these challenges. This collection of papers reflects the evolving local and global contexts and emerging priorities for health promotion. Insofar as promoting health is one of the three strategic priorities for the WHO’s 13th General Programme of Work, the papers point to the evolution of our paradigm about health—i.e., shifting from disease to risk factors to determinants, and from individual, through social to planetary health and wellbeing. Research, policy and practice in health promotion will need to similarly evolve to keep up with these emerging health challenges and changing contexts.

## DISCLAIMER

The authors alone are responsible for the views expressed in this article and they do not necessarily represent the views, decisions or policies of the institutions with which they are affiliated.
